# BDL: transformer-based super-resolution network for degraded underground coal mine images

**DOI:** 10.1038/s41598-026-48248-1

**Published:** 2026-04-13

**Authors:** Tao Hu, Jinbo Qiu, Xiang Cheng

**Affiliations:** 1 State Key Laboratory of Intelligent Coal Mining and Strata Control, Shanghai, 200030 China; 2 China Coal Technology and Engineering Group Shanghai Co., Ltd, Shanghai, 200030 China; 3 The Chinese Antarctic Center of Surveying and Mapping, Wuhan University, Wuhan, 430079 China

**Keywords:** Underground coal mine images, Super-resolution, Transformer-based network, Bidirectional adaptive interaction module (BAIM), Dual-group feedforward network (DGFN), Energy science and technology, Engineering, Environmental sciences, Mathematics and computing, Solid Earth sciences

## Abstract

Underground coal mine images often suffer from severe blurring and low-resolution degradation due to harsh lighting, dust, and machinery motion, which hinder accurate visual inspection and automated analysis. This study proposes a transformer-based super-resolution (SR) network that integrates local convolution with adaptive interaction mechanisms for effective local–global feature modeling. The network employs a hierarchical architecture consisting of shallow feature extraction, cascaded spatial and channel transformer blocks, and a reconstruction module. Each transformer block incorporates a bidirectional adaptive interaction module (BAIM) to fuse convolutional local features with transformer-based global representations through adaptive reweighting in both spatial and channel dimensions. A dual-group feedforward network (DGFN) decouples channel feature preservation from spatial information enhancement, while cross-group interactions ensure balanced channel modeling and spatial perception without information loss. Additionally, a local convolution block (LCB) with SE-based channel weighting is used to restore fine-grained details. Extensive experiments on both a dedicated coal mine dataset and public benchmarks demonstrate that the proposed method consistently outperforms existing state-of-the-art (SOTA) SR approaches. Specifically, for ×2 super-resolution, it achieves a PSNR/SSIM of 32.07/0.9688 on the coal mine dataset, improving over the previous best by 0.59 dB and 0.0036, respectively. For ×4 super-resolution, it attains 28.10/0.8836, surpassing the previous best by 0.24 dB and 0.0013. Similar improvements are observed on public datasets, confirming the method’s effectiveness in both general and challenging industrial scenarios.

## Introduction

Coal remains the primary energy source worldwide and plays a crucial role in national economic and social development. With the implementation of the “carbon peaking and carbon neutrality” strategy, the coal industry is rapidly transitioning toward intelligent and green mining^1^. In this context, intelligent and unmanned mining technologies have become key approaches for improving production safety and efficiency. High-quality underground mine images provide essential visual information for intelligent perception systems in coal mines^2^. However, due to complex underground conditions such as uneven illumination, dust interference, and low-light environments, the images captured by monitoring systems often exhibit low resolution, insufficient brightness, and blurred details^3^. These issues significantly degrade visual perception and limit the performance of downstream intelligent analysis systems.

Image super-resolution (SR), which aims to reconstruct high-resolution images from low-resolution inputs, has become an effective solution for improving visual quality in degraded imaging conditions^4^. Early SR methods mainly relied on interpolation- and reconstruction-based approaches, such as bicubic interpolation and projection onto convex sets (POCS). Although these traditional techniques are computationally efficient, they often fail to recover high-frequency details in complex scenes^5^. With the rapid development of deep learning, convolutional neural networks (CNNs) have significantly improved SR performance by learning end-to-end mappings between low-resolution and high-resolution images^6^. However, CNN-based models are inherently limited by their local receptive fields, which restrict their ability to capture long-range dependencies and global contextual information^7^. Recently, transformer architectures have demonstrated strong capability in modeling global contextual relationships through self-attention mechanisms and have been successfully applied to image restoration and super-resolution tasks^8^. Nevertheless, directly applying existing transformer-based SR models to underground coal mine images remains challenging. On the one hand, transformer architectures may struggle to capture fine-grained local features, leading to insufficient edge and texture reconstruction. On the other hand, complex mine imaging conditions increase the difficulty of feature extraction and representation. Moreover, many existing methods lack effective mechanisms for adaptively integrating global and local features, which limits their applicability to underground coal mine scenarios^9^.

This paper proposes a BDL-based super-resolution framework tailored for underground coal mine images. BDL stands for bidirectional adaptive interaction module (BAIM), dual-group feedforward network (DGFN), and local convolution block (LCB). The proposed approach introduces the BAIM to enable dynamic interaction between spatial and channel features, facilitating effective fusion of local convolutional details and global transformer representations. In addition, the DGFN is designed to decouple spatial and channel feature modeling, improving the preservation of high-frequency information during reconstruction. Furthermore, the integration of a LCB with channel attention enhances texture and edge recovery. The main contributions of this work are summarized as follows:

1. We propose BAIM to integrate global and local features through adaptive reweighting in both spatial and channel dimensions, improving the Transformer’s ability to model complex image information.
2. We introduce DGFN, which separates channel feature preservation from spatial enhancement, using cross-group interaction to balance channel modeling and spatial awareness, avoiding common information loss in traditional gating.
3. We present LCB, a local-global feature architecture combining multi-level convolutions and channel weighting with SE attention, significantly enhancing image detail restoration.

The remainder of this paper is organized as follows. Section 2 reviews related work SR. Section 3 describes the proposed framework in detail. Section 4 presents experimental results and comparative analysis. Section 5 provides further discussion, and Sect. 6 concludes the paper.

## Related work

Image enhancement techniques aim to improve visual quality under challenging illumination conditions. Traditional histogram equalization methods often suffer from over-enhancement or brightness distortion when applied to low-light images. To address these limitations, several improved approaches have been proposed. Wu et al.^10^ introduced a hybrid saliency map-based enhancement method to improve image contrast while maintaining brightness consistency. Yang et al.^11^ proposed a dual-branch low-light enhancement algorithm with brightness constraints to balance enhancement performance and brightness preservation. Wang et al.^12^ further designed a multi-module fusion framework based on the Retinex physical model combined with deep networks, integrating multi-scale guidance blocks and global spatial attention to improve image quality in low-light conditions.

Deep learning has significantly advanced the development of super-resolution methods. Zhou et al.^13^ proposed a robust SR compressive sensing approach based on a two-timescale alternating MAP estimation framework. Wang et al.^14^ introduced a compressed adaptive-sampling-rate sensing method using an overcomplete ridgelet dictionary to dynamically allocate sampling rates according to image block sparsity. Wu et al.^15^ proposed a lightweight SR network using fully 1 × 1 convolutional layers combined with spatial shift operations to reduce model parameters. More advanced CNN architectures have also been proposed to improve feature representation. Xie et al.^16^ developed a multi-scale feedback residual network to enhance information flow between layers. Kaur and Singh^17^ introduced enhanced pyramidal residual networks to improve structural detail reconstruction. Xie et al.^18^ further proposed MEEAFusion, which integrates multi-scale edge enhancement with a joint attention mechanism to improve structural integrity during image reconstruction. Generative adversarial networks (GANs) have also been widely applied in SR tasks. Park et al.^19^ proposed NeXtSRGAN to improve texture realism and structural fidelity. Zhang et al.^20^ developed an improved GAN-based SR model for higher reconstruction accuracy. Yang et al.^21^ designed a GAN-based SR algorithm specifically for underground coal mine video images. Wang et al.^22^ combined SR reconstruction with machine learning in the SAM-EMSR-GAN + CatBoost framework for multispectral coal gangue recognition. Zou et al.^23^ proposed a lightweight GAN-based SR approach that significantly improves image clarity and detail representation.

Transformer architectures have recently demonstrated strong capability in modeling long-range dependencies for vision tasks. The transformer was first proposed by Vaswani et al.^24^, introducing the self-attention mechanism for sequence modeling. Dosovitskiy et al.^25^ extended this concept to visual tasks through the Vision Transformer (ViT). To improve computational efficiency, Chen et al.^26^ proposed the Swin Transformer, which employs a shifted window attention mechanism. Zhang et al.^27^ also proposed an efficient hybrid CNN–Transformer SR framework that integrates local feature extraction with global contextual modeling. Zhou et al.^28^ introduced windowed positional self-attention (WPSA) in early Transformer layers to simulate convolution’s local inductive bias, enhancing local feature capture. Li et al.^29^ achieved a similar goal in MLPs by designing parallel multi-scale branches to fuse local and global information across receptive fields. Zhou et al.^30^ proposed a spatial reduction dual-attention (SRDA) mechanism, improving feature representation and reducing computational complexity through self-check similarity between key-value pairs. Ma et al.^31^ integrated Mamba’s state-space model with linear attention, balancing local and global context while addressing the quadratic complexity of Transformers. Ma et al.^32^ improved autoencoder sensitivity to subtle anomalies by introducing patch-level aggregation and cross-dimensional scoring. Xie et al.^33^ proposed an asymmetric convolutional modulation network (ACMN) for efficient image super-resolution, which combines large kernel design, asymmetric convolution, and a channel shuffle feedforward network to improve reconstruction performance while reducing model complexity. Although these transformer-based SR methods show promise, they are often challenged by the specific complexities of underground coal mine imagery. Coal mine images exhibit unique degradation characteristics such as uneven lighting, dust, and low-light conditions, which make traditional transformer architectures struggle to capture fine-grained local features. Moreover, existing methods tend to focus on modeling global context but fail to effectively address how to integrate local features and global information, a problem that is particularly pronounced in complex coal mine environments. To overcome these challenges, this paper proposes a BDL-based super-resolution framework, integrating adaptive interaction mechanisms to effectively address the complexities of underground coal mine imagery.

## Proposed method

### Overall Model Architecture

**Fig. 1:**
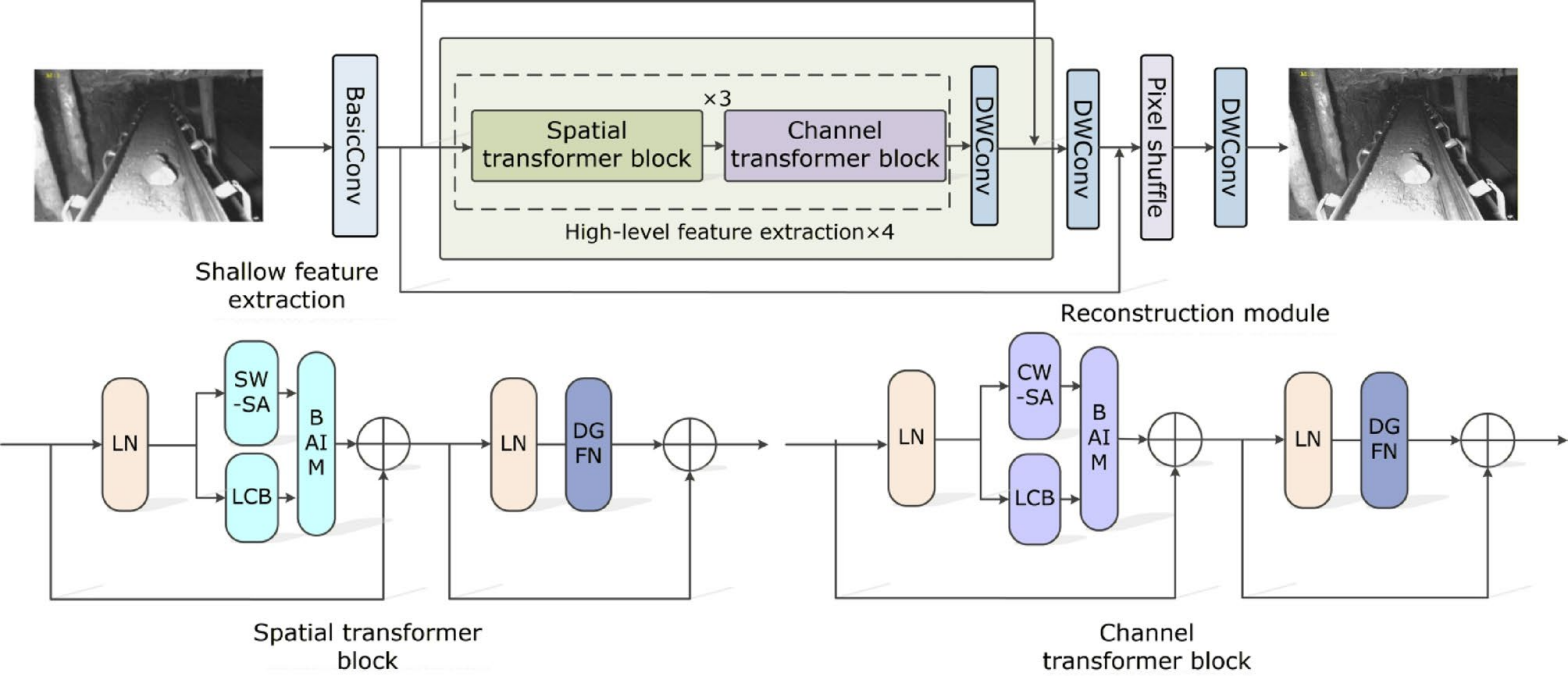
Overall architecture of the reconstruction model.

To enhance the reconstruction quality and detail restoration of low-quality mine images, this paper proposes a transformer architecture that integrates local convolution with bidirectional adaptive interaction module (BAIM). The overall model employs a three-stage progressive construction strategy, consisting of three main components: a shallow feature extraction module, a high-level feature extraction module, and a reconstruction module. The overall network architecture is depicted in Fig. 1.

Given a low-resolution input image, the model first utilizes a basic convolutional layer to extract shallow features. This layer consists of convolution, batch normalization, and activation functions. The extracted shallow features primarily contain low-level visual cues such as image contours, edges, color, texture, and shape. These shallow features are then fed into the high-level feature extraction module for further processing. The overall network contains four high-level feature extraction modules, each high-level feature extraction module comprises three dual spatial transformer blocks (DSTBs) and dual channel transformer blocks (DCTBs). As illustrated in Fig. 1, the DSTB includes layer normalization (LN), spatial window self-attention (SW-SA), local convolution block (LCB), BAIM, and DGFN. The DCTB consists of LN, channel-wise self-attention (CW-SA), LCB, BAIM, and DGFN. By alternately integrating the SW-SA and CW-SA modules, the model enables collaborative modeling of spatial and channel features. Specifically, SW-SA focuses on modeling local spatial context, enhancing the spatial representation of feature maps, while CW-SA captures global dependencies across channels, effectively expanding the receptive field. This, in turn, improves SW-SA’s ability to capture spatial information. The complementary integration of SW-SA and CW-SA not only enriches the model’s multi-dimensional feature representation capabilities but also enhances its ability to model complex scenarios.These modules extract more abstract and complex representations, such as object semantics, scene understanding, and contextual relationships, which are crucial for deep interpretation and analysis of image content. Subsequently, the aggregated shallow and high-level features are passed to the reconstruction module. In the reconstruction stage, depthwise separable convolution (DW-Conv) is first applied to capture fine-grained local features while reducing parameter overhead and improving feature representation efficiency. Then, pixel shuffle is used for spatial upsampling, transforming low-resolution features into high-resolution images. Finally, another DW-Conv layer is employed to enhance details and suppress noise in the upsampled features, thereby improving the structural fidelity and overall quality of the reconstructed image.

### Local Convolution Block

**Fig. 2:**
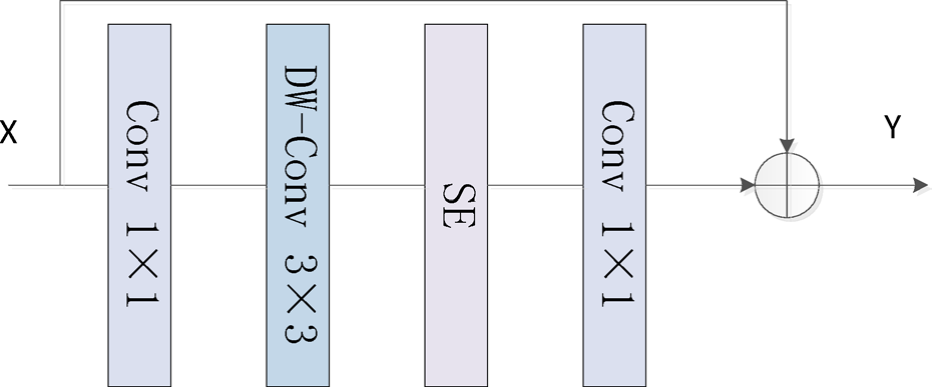
Local convolution block network structure.

The local convolution block (LCB) is designed to efficiently extract local spatial features while enhancing the model’s representational capacity and non-linear modeling ability^34^. This module consists of four main components: a 1 × 1 convolution, a 3 × 3 DW-Conv, a squeeze-and-excitation (SE) attention mechanism, and another 1 × 1 convolution. A residual connection is employed to directly add the input to the output, thereby facilitating information flow and improving the stability of gradient backpropagation. As illustrated in Fig. 2.

Given an input feature map$\:\:\mathrm{X}\in\:{R}^{H\times\:W\times\:C}$, where *H* is the height, *W* the width, and C is the number of channels, the module operates as follows:

1 $$\:{\mathrm{X}}_{1}={\mathrm{C}\mathrm{o}\mathrm{n}\mathrm{v}}_{1\times\:1}\left(\mathrm{X}\right)$$

2 $$\:{\mathrm{X}}_{2}={\mathrm{D}\mathrm{W}\mathrm{C}\mathrm{o}\mathrm{n}\mathrm{v}}_{3\times\:3}\left({\mathrm{X}}_{1}\right)$$

3 $$\:\mathrm{Z}=\frac{1}{\mathrm{H}.\mathrm{W}}\sum\:_{\mathrm{i}=1}^{\mathrm{H}}\sum\:_{\mathrm{j}=1}^{\mathrm{W}}{\mathrm{X}}_{2}(\mathrm{i},\mathrm{j})$$

4 $$\:\mathrm{S}=\mathrm{S}\mathrm{i}\mathrm{g}\mathrm{m}\mathrm{o}\mathrm{i}\mathrm{d}({\mathrm{W}}_{\mathrm{m}}.\mathrm{R}\mathrm{e}\mathrm{L}\mathrm{U}\left({\mathrm{W}}_{\mathrm{n}}.\mathrm{Z}\right))$$

5 $$\:{\mathrm{X}}_{3}={\mathrm{X}}_{2}\odot\:\mathrm{S}$$

6 $$\:{\mathrm{X}}_{4}={\mathrm{C}\mathrm{o}\mathrm{n}\mathrm{v}}_{1\times\:1}\left({\mathrm{X}}_{3}\right)$$

7 $$\:\mathrm{Y}=\mathrm{X}+{\mathrm{X}}_{4}$$

Where $\:{W}_{n}\in\:{R}^{\frac{C}{r}\times\:C}$ and $\:{W}_{m}\in\:{R}^{C\times\:\frac{C}{r}}$ are weight matrices, “⊙” typically denotes element-wise multiplication, $\:{X}_{1}$ to $\:{X}_{4}$ represent the outputs of each sub-module within the network architecture. S denotes the channel-wise attention weights, and Y is the final output. This module effectively integrates the local modeling capability of convolutional operations with the global contextual modeling strength of the attention mechanism, thereby enhancing feature representation while maintaining computational efficiency.

### Bidirectional adaptive interaction module

**Fig. 3:**
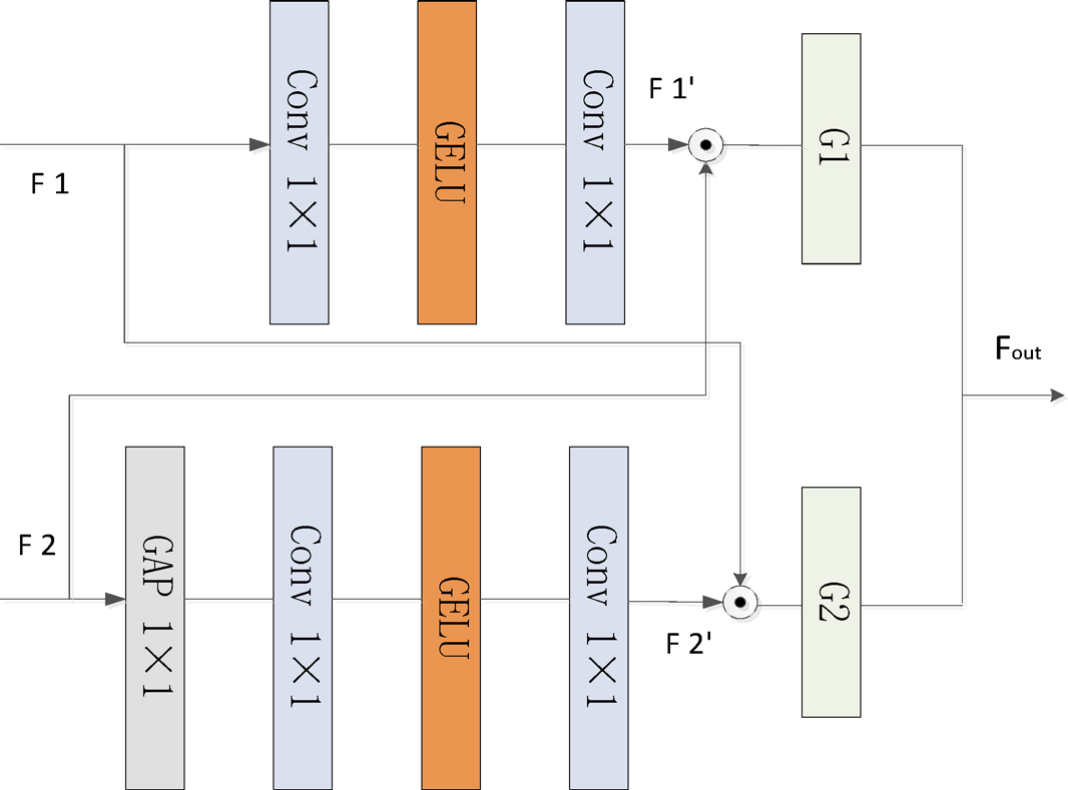
Bidirectional adaptive interaction module.

Although alternating between SW-SA and CW-SA enables the capture of spatial and channel features separately^35^, a single self-attention module still lacks the ability to fully exploit and coordinate multi-dimensional features. To address the insufficient integration of global and local features in existing transformer architectures, this paper proposes a bidirectional adaptive interaction module (BAIM), as illustrated in Fig. 3.

Unlike traditional self-attention mechanisms that focus solely on long-range dependencies, this module adaptively reweights dual-branch features along the spatial or channel dimension based on the type of self-attention mechanism. Given two input features $\:{F}_{1}\in\:{R}^{H\times\:W\times\:C}$ and $\:{F}_{2}\in\:{R}^{H\times\:W\times\:C}$, the operation is defined as follows:

8 $$\:{\mathrm{F}1}^{{\prime\:}}={\mathrm{W}}_{2}\mathrm{G}\mathrm{E}\mathrm{L}\mathrm{U}\left({\mathrm{W}}_{1}{F}_{1}\right)$$

9 $$\:{\mathrm{F}2}^{{\prime\:}}={\mathrm{W}}_{4}\mathrm{G}\mathrm{E}\mathrm{L}\mathrm{U}\left({\mathrm{W}}_{3}{W}_{H}^{GP}{F}_{2}\right)$$

10 $$\:{\upalpha\:}1=\mathrm{S}\mathrm{i}\mathrm{g}\mathrm{m}\mathrm{o}\mathrm{i}\mathrm{d}({\mathrm{C}\mathrm{o}\mathrm{n}\mathrm{v}}_{1\times\:1}\left({\mathrm{F}1}^{{\prime\:}}\odot\:\mathrm{F}2\right)+{\mathrm{C}\mathrm{o}\mathrm{n}\mathrm{v}}_{1\times\:1}\left({\mathrm{F}2}^{{\prime\:}}\odot\:\mathrm{F}1\right))$$

11 $$\:{\upalpha\:}2=1-{\upalpha\:}1$$

12 $$\:{\mathrm{F}}_{\mathrm{o}\mathrm{u}\mathrm{t}}=\left({\upalpha\:}1{(\mathrm{F}1+\mathrm{F}1}^{{\prime\:}}\right)+{\upalpha\:}2{(\mathrm{F}2+\mathrm{F}2}^{{\prime\:}}\left)\right){\mathrm{W}}_{2}$$

Where, $\:{W}_{1}$ and $\:{W}_{3}$​ are convolutional kernels for dimensionality reduction, while $\:{W}_{2}$​ and $\:{W}_{4}$​​ are convolutional kernels for dimensionality expansion. GELU refers to the Gaussian Error Linear Unit activation function. $\:{W}_{H}^{GP}$ denotes weighted global pooling, “⊙” represents element-wise multiplication, $\:{\upalpha\:}1$​ and $\:{\upalpha\:}2$ are learnable weighting parameters, and $\:{F}_{out}$ is the output feature after adaptive fusion.

### Dual-gated feed-forward network

 The feed-forward network (FFN), as one of the core components of the trans-former architecture, plays a critical role in feature space mapping and nonlinear transformation^36^. Typically designed with a two-layer linear transformation com-bined with a nonlinear activation function, the FFN significantly enhances the model’s representation capacity while maintaining the dimensionality of the feature tensor. Through position-independent feature processing, the FFN is capable of modeling more complex feature interactions, complementing the functionality of the self-attention mechanism. This dual-branch architecture effectively strengthens the network’s ability to capture multi-level features, thereby enabling deeper semantic understanding of the input.

However, the conventional FFN architecture exhibits notable limitations: its computational resources are predominantly focused on channel-wise transformations, which leads to insufficient modeling of local spatial features in tasks sensitive to spatial information, such as super-resolution. To address this issue, existing studies have primarily focused on structural optimization—typically by introducing DW-Conv after channel expansion layers to enhance spatial feature extraction, often coupled with spatial gating mechanisms to improve spatial representation. Nevertheless, such en-hancements frequently suffer from channel dimension compression, limiting the net-work’s modeling capacity in high-dimensional feature spaces. To overcome these challenges, this paper introduces DGFN, which incorporates a self-gating unit into the spatial feature branch to effectively restore channel information lost during the gating process. Additionally, a gating alignment strategy is designed to ensure coordinated operation with the cross-gating mechanism in the parallel channel branch. The formulation of DGFN is as follows:

13 $$\:{\mathrm{X}}_{\mathrm{e}\mathrm{x}\mathrm{p}1}=\mathrm{E}\mathrm{x}\mathrm{p}1\left({\mathrm{X}}_{\mathrm{i}\mathrm{n}\mathrm{p}\mathrm{u}\mathrm{t}}\right)$$

14 $$\:{\mathrm{X}}_{\mathrm{e}\mathrm{x}\mathrm{p}2}=\mathrm{D}\mathrm{W}\mathrm{C}\mathrm{o}\mathrm{n}\mathrm{v}\left({\mathrm{X}}_{\mathrm{e}\mathrm{x}\mathrm{p}1}\right)$$

15 $$\:{\mathrm{X}}_{1},{\mathrm{X}}_{2}=\mathrm{S}\mathrm{p}\mathrm{l}\mathrm{i}\mathrm{t}\left({\mathrm{X}}_{\mathrm{e}\mathrm{x}\mathrm{p}2}\right)$$

16 $$\:{\mathrm{X}}_{\mathrm{s}-\mathrm{g}\mathrm{a}\mathrm{t}\mathrm{e}}={\mathrm{X}}_{1}.\mathrm{G}\mathrm{e}\mathrm{l}\mathrm{u}\left({\mathrm{X}}_{1}\right)$$

17 $$\:{\mathrm{X}}_{\mathrm{c}-\mathrm{g}\mathrm{a}\mathrm{t}\mathrm{e}}={\mathrm{X}}_{2}.\mathrm{G}\mathrm{e}\mathrm{l}\mathrm{u}\left({\mathrm{X}}_{1}\right)$$

18 $$\:{\mathrm{X}}_{\mathrm{o}\mathrm{u}\mathrm{t}\mathrm{p}\mathrm{u}\mathrm{t}}=\mathrm{S}\mathrm{q}\mathrm{z}\left(\mathrm{C}\mathrm{a}\mathrm{t}\left({\mathrm{X}}_{\mathrm{s}-\mathrm{g}\mathrm{a}\mathrm{t}\mathrm{e}},{\mathrm{X}}_{\mathrm{c}-\mathrm{g}\mathrm{a}\mathrm{t}\mathrm{e}}\right)\right)$$

**Fig. 4:**
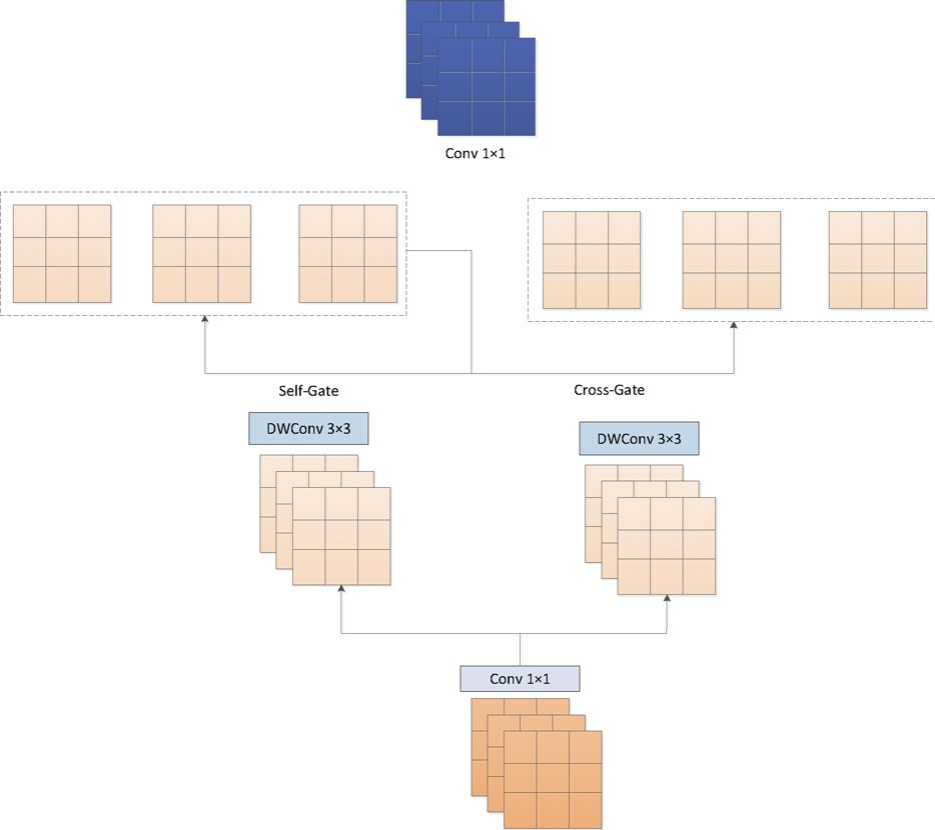
Structure of the dual-gated feed-forward network.

$\:{X}_{input}, {X}_{output}\in\:{R}^{H\times\:W\times\:C}$ denote the input and output features, respectively, and $\:{X}_{(.)}$ represents intermediate features. Exp1 and Sqz refer to the channel expansion and squeezing operations, respectively. DWConv is a depthwise separable convolution with a 3 × 3 kernel used for spatial information extraction. The structure is illustrated in Fig. 4.

## Experimental results and analysis

### Datasets

The coal mine dataset used in the experiments consists of images from the publicly available CMUID dataset^37^ and images collected from underground coal mines. The collected images were sourced from real underground mine and were carefully screened to ensure image quality and scene diversity. The images were downsampled by factors of 2× and 4× to construct paired high-resolution (HR) and low-resolution (LR) images for model training. In total, 1000 valid samples were obtained, including 600 images from the CMUID dataset and 400 images collected from real underground mine. Among them, 950 images were used for training and 50 images were used for testing, and the training and testing sets were strictly separated to avoid data leakage during model evaluation. In addition, the publicly available DIV2K dataset was used for training, and performance comparisons were conducted on benchmark datasets, including Set5 dataset^38^, Set14 dataset^39^, and Urban100 dataset^40^.

## Experimental environment and parameter settings

To validate the effectiveness and reliability of the proposed network model, experiments were conducted under consistent conditions. The experimental platform was a high-performance computing workstation equipped with an Intel Core i7-12700 processor, 64 GB of RAM, and an NVIDIA RTX 3090 GPU, running the 64-bit version of Windows 10. The software environment included Python 3.8 and PyTorch version 1.13.0 as the deep learning framework. The experimental environment is shown in Table 1.

**Table 1:** Experimental environment.

Configuration	Parameters
Deep learning framework	Pytorch 1.13.0 + python 3.8.0
Operating system	Windows10
GPU	NVIDIA GeForce RTX 3090
CPU	Intel(R) Core(TM) i7-12700@2.10 GHz

In this study, to accelerate training, the training images were cropped into overlapping patches of size 256 × 256. The training was performed for 50,000 iterations with a batch size of 16. The Adam optimizer (with parameters β₁ = 0.9 and β₂ = 0.99) was employed, and the model was optimized by minimizing the L1 loss. The initial learning rate was set to 5 × 10⁻³ and was halved at iterations 15,000, 25,000, 35,000, and 45,000, respectively.

## Evaluation metrics

To quantitatively assess the performance of the image reconstruction algorithms, this paper employs peak signal-to-noise ratio (PSNR) and structural similarity index measure (SSIM) as the primary evaluation metrics^41^. PSNR objectively evaluates image fidelity by calculating the ratio between the maximum possible signal power and the noise power in the reconstructed image compared to the original image. SSIM assesses image similarity from three aspects: luminance, contrast, and structural information. The definitions are as follows:

19 $$\:\mathrm{P}\mathrm{S}\mathrm{N}\mathrm{R}(\mathrm{I},\widehat{\mathrm{I}})=10.{\mathrm{l}\mathrm{o}\mathrm{g}}_{10}\left(\frac{{255}^{2}}{\mathrm{M}\mathrm{S}\mathrm{E}(\mathrm{I},\widehat{\mathrm{I}})}\right)$$

20 $$\:\mathrm{S}\mathrm{S}\mathrm{I}\mathrm{M}(\mathrm{I},\widehat{\mathrm{I}})=\frac{(2{\mathrm{u}}_{\mathrm{I}}{\mathrm{u}}_{\widehat{\mathrm{I}}}+{\mathrm{C}}_{1})(2{{\upsigma\:}}_{\mathrm{I}\widehat{\mathrm{I}}}+{\mathrm{C}}_{2})}{({{\mathrm{u}}_{\mathrm{I}}}^{2}+{{\mathrm{u}}_{\widehat{\mathrm{I}}}}^{2}+{\mathrm{C}}_{1})({{{\upsigma\:}}_{\mathrm{I}}}^{2}+{{{\upsigma\:}}_{\widehat{\mathrm{I}}}}^{2}+{\mathrm{C}}_{2})}$$

Where, $\:I$ and $\:\widehat{I}$ represent the original image and the reconstructed image, respectively, and $\:MSE(I,\widehat{I})$ denotes the mean squared error between them. µ represents the mean of the image, σ denotes the standard deviation, $\:{\sigma\:}_{I\widehat{I}}$​ is the covariance, $\:{C}_{1}$​ and $\:{C}_{2}$ are stability constants.

## Experimental results

To more comprehensively verify the practical effectiveness of the proposed super-resolution reconstruction method for underground coal mine images, in addition to employing objective metrics such as PSNR and SSIM, a subjective visual comparison was further conducted, focusing on edge preservation and texture detail restoration, so as to reflect the actual visual quality of the reconstructed images. In this section, three representative images of coal mining faces and roadways in underground mines were selected as test samples. Under a scaling factor of 4, the proposed method was compared with Bicubic^42^, SRCNN^15^, VDSR^43^, EDSR^44^, SwinIR^45^, and DAT^35^.

**Fig. 5:**
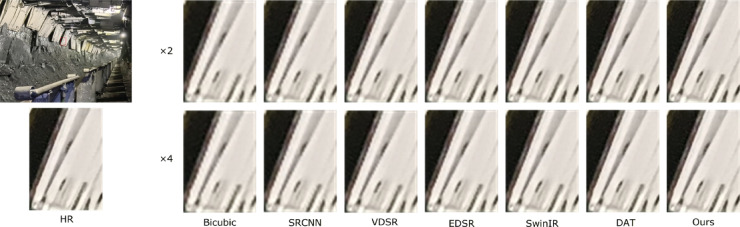
Comparison of reconstruction results at ×4 scale for different methods in coal mine underground scene 1.

**Fig. 6:**
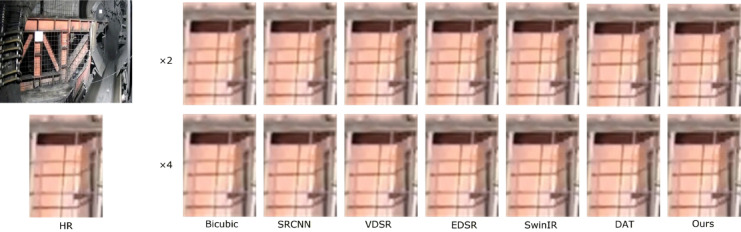
Comparison of reconstruction results at ×4 scale for different methods in coal mine underground scene 2.

**Fig. 7:**
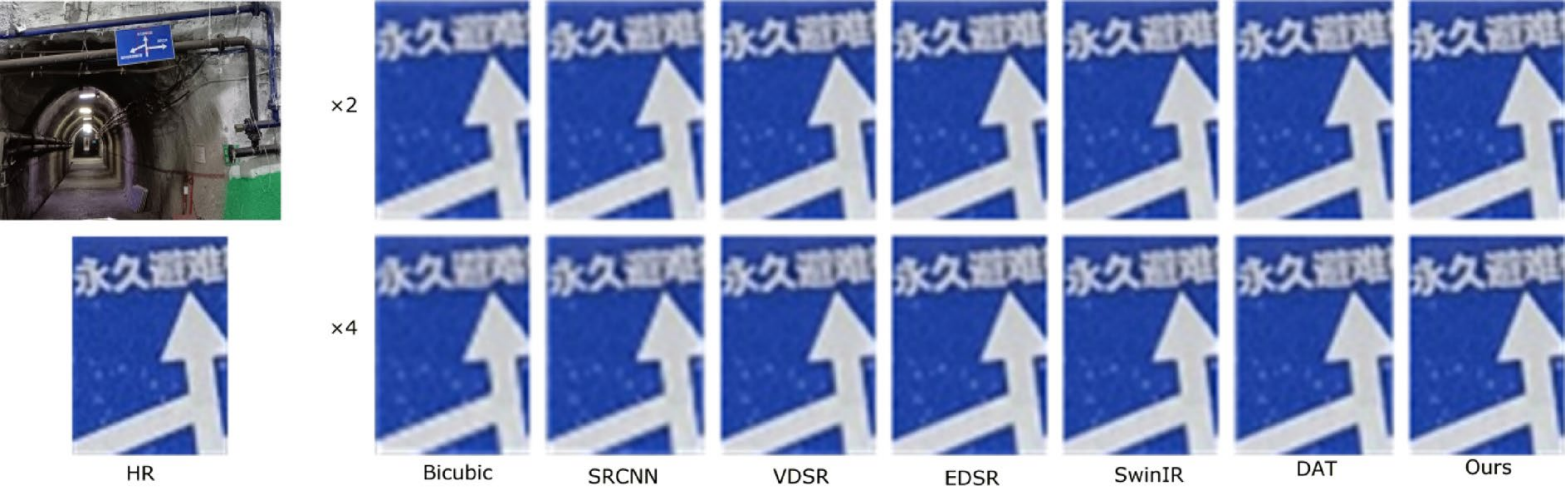
Comparison of reconstruction results at ×4 scale for different methods in coal mine underground scene 3.

**Fig. 8:**
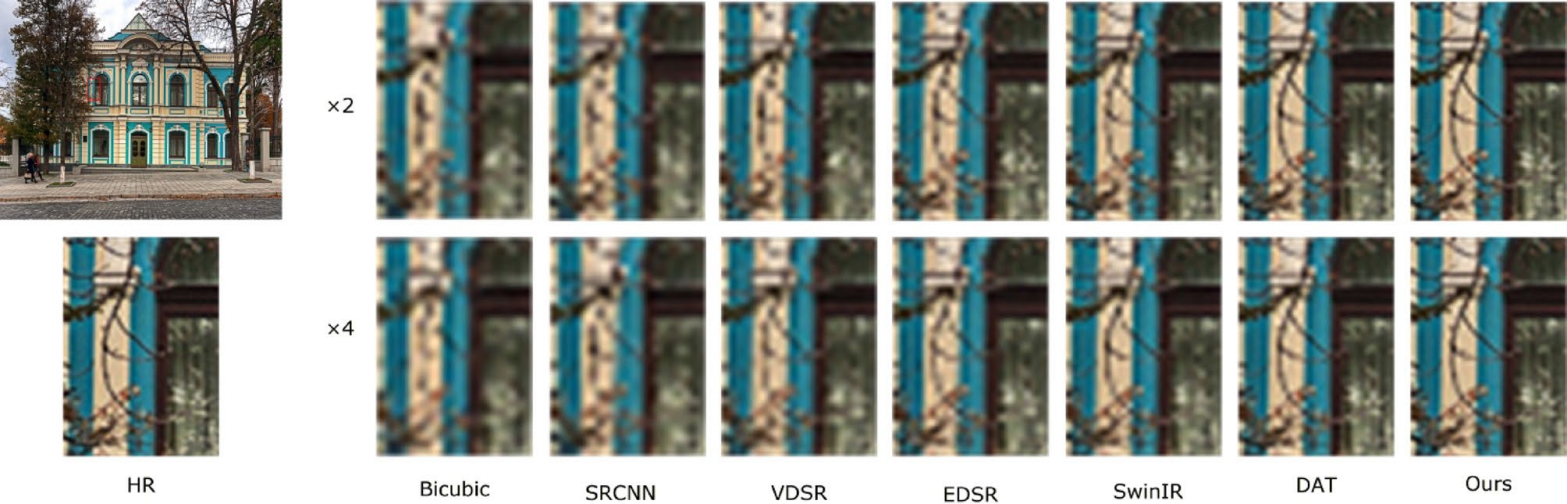
Comparison of reconstruction results at ×4 scale for different methods in coal mine underground scene 4.

During the experiments, low-resolution images downsampled by factors of ×2 and ×4 were input into the pre-trained models for super-resolution reconstruction. The reconstructed results for both settings are presented in Figs. 5, 6, 7 and 8, and are evaluated in terms of edge contour clarity, texture detail fidelity, and noise suppression capability. It can be observed that the proposed method more effectively restores equipment structural details and fine textures in complex underground scenes, producing images with higher visual clarity and stronger structural coherence compared to other methods. In particular, the proposed method consistently achieves better reconstruction results than competing approaches, especially in regions with complex textures and structural edges. Moreover, the reconstruction performance under the ×2 downsampling setting is noticeably better than that under ×4, as the milder degradation allows for more accurate recovery of high-frequency details.

To thoroughly assess the objective performance metrics of the aforementioned super-resolution reconstruction methods on coal mine images, four representative mine images shown in Figs. 5, 6, 7 and 8 were selected as test samples. Reconstruction comparisons were conducted using these methods under scaling factors of 2 and 4. The PSNR and SSIM values for each method were computed, with the results presented in Table 2.

**Table 2:** Results of various SR algorithms for 3 specific scene images at scale of×2 and×4.

Scale	Method	Figure 5	Figure 6	Figure 7	Figure 8
		PSNR/SSIM	PSNR/SSIM	PSNR/SSIM	PSNR/SSIM
	Bicubic	28.15/0.9321	27.89/0.9287	28.34/0.9345	28.51/0.9362
×2	SRCNN	30.26/0.9623	30.08/0.9589	30.47/0.9638	30.62/0.9653
	VDSR	30.52/0.9648	30.31/0.9607	30.63/0.9652	30.78/0.9665
	EDSR	30.88/0.9684	30.70/0.9648	30.92/0.9695	31.19/0.9708
	SwinIR	31.12/0.9720	30.95/0.9686	31.28/0.9703	31.52/0.9723
	DAT	31.45/0.9765	31.32/0.9721	31.67/0.9783	32.46/0.9807
	Ours	32.02/0.9801	31.84/0.9758	32.35/0.9812	33.72/0.9853
	Bicubic	24.73/0.8123	24.55/0.8067	24.88/0.8154	25.06/0.8172
×4	SRCNN	25.94/0.8432	25.76/0.8379	26.12/0.8465	26.25/0.8483
	VDSR	26.21/0.8517	25.81/0.8426	26.25/0.8493	26.38/0.8514
	EDSR	26.31/0.8590	25.98/0.8509	26.56/0.8562	26.72/0.8582
	SwinIR	26.73/0.8617	26.25/0.8545	26.98/0.8645	27.15/0.8663
	DAT	26.87/0.8621	26.63/0.8574	27.05/0.8659	27.25/0.8675
	Ours	27.32/0.8709	26.48/0.8656	27.46/0.8768	27.62/0.8785

The experimental results show that the Bicubic method produces reconstructed images with noticeable detail blurring, failing to meet the requirements for restoring underground equipment and structural details. Although SRCNN improves image quality to some extent, the reconstructed images still lack sufficient texture features and exhibit limited detail richness. Both the DAT method and the proposed method demonstrate better performance in preserving image textures and edges, effectively restoring image structures in complex scenes. Notably, the proposed method achieves higher PSNR and SSIM values across all test images, indicating its superior capability in reconstruction accuracy and perceptual quality.

To evaluate the impact of each module within the proposed model on super-resolution reconstruction performance, an ablation study was conducted on the coal mine image test set under a 2× scaling task. The experimental results are presented in Table 3. This table illustrates the effects of four different module combinations (LCB, BAIM, and DGFN) on image reconstruction performance, measured by PSNR and SSIM. The configurations are as follows: Model A (containing only LCB), Model B (containing LCB and BAIM), Model C (containing BAIM and DGFN), and Model D (integrating LCB, BAIM, and DGFN).

**Table 3:** The impact of different modules on model performance at ×2 scale.

Model	LCB	BAIM	DGFN	Params (M)	FLOPs (G)	PSNR	SSIM
A	√	×	×	10.85	195.32	30.95	0.9463
B	√	√	×	10.95	198.96	31.47	0.9501
C	×	√	√	11.03	197.18	31.68	0.9512
D	√	√	√	11.08	198.56	32.07	0.9688

**Table 4:** Effect of BAIM analysis.

Model	Fusion Type	PSNR	SSIM
E	Add	31.28	0.9483
F	Concat + Conv	31.53	0.9507
G	BAIM	32.07	0.9688

**Table 5:** Effect of FFN design analysis.

Model	FFN Type	PSNR	SSIM
H	Standard FFN	31.42	0.9497
I	DWConv-FFN	31.75	0.9542
J	DGFN	32.07	0.9688

**Table 6:** Effect of bidirectional analysis.

Model	Spatial Branch	Channel Branch	PSNR	SSIM
K	√	×	31.65	0.9509
L	×	√	31.72	0.9535
M	√	√	32.07	0.9688

The experimental results in Table 3 indicate that the model performance steadily improves as the number of incorporated modules increases. The baseline Model A, which includes only the LCB module, achieves a PSNR of 30.95 and an SSIM of 0.9463, demonstrating that the LCB module contributes positively to performance enhancement. By further introducing the BAIM module in Model B, the PSNR increases to 31.47 and the SSIM to 0.9501, indicating that BAIM effectively enhances the extraction of image structural information. Although Model C excludes the LCB module, it combines BAIM and DGFN, achieving a PSNR of 31.68 and an SSIM of 0.9512, which slightly surpasses Model B, reflecting the strong capability of the DGFN module in jointly modeling global and detailed information. The final Model D integrates all three modules, exhibiting the best performance with a PSNR of 32.07 and an SSIM of 0.9688, fully validating the complementary and synergistic effects among these modules. The results in Table 3 show that the improvements in PSNR and SSIM are achieved with only marginal increases in parameters (Params) and FLOPs, indicating that the performance gains mainly stem from the proposed architectural design rather than model scale.

**Fig. 9:**
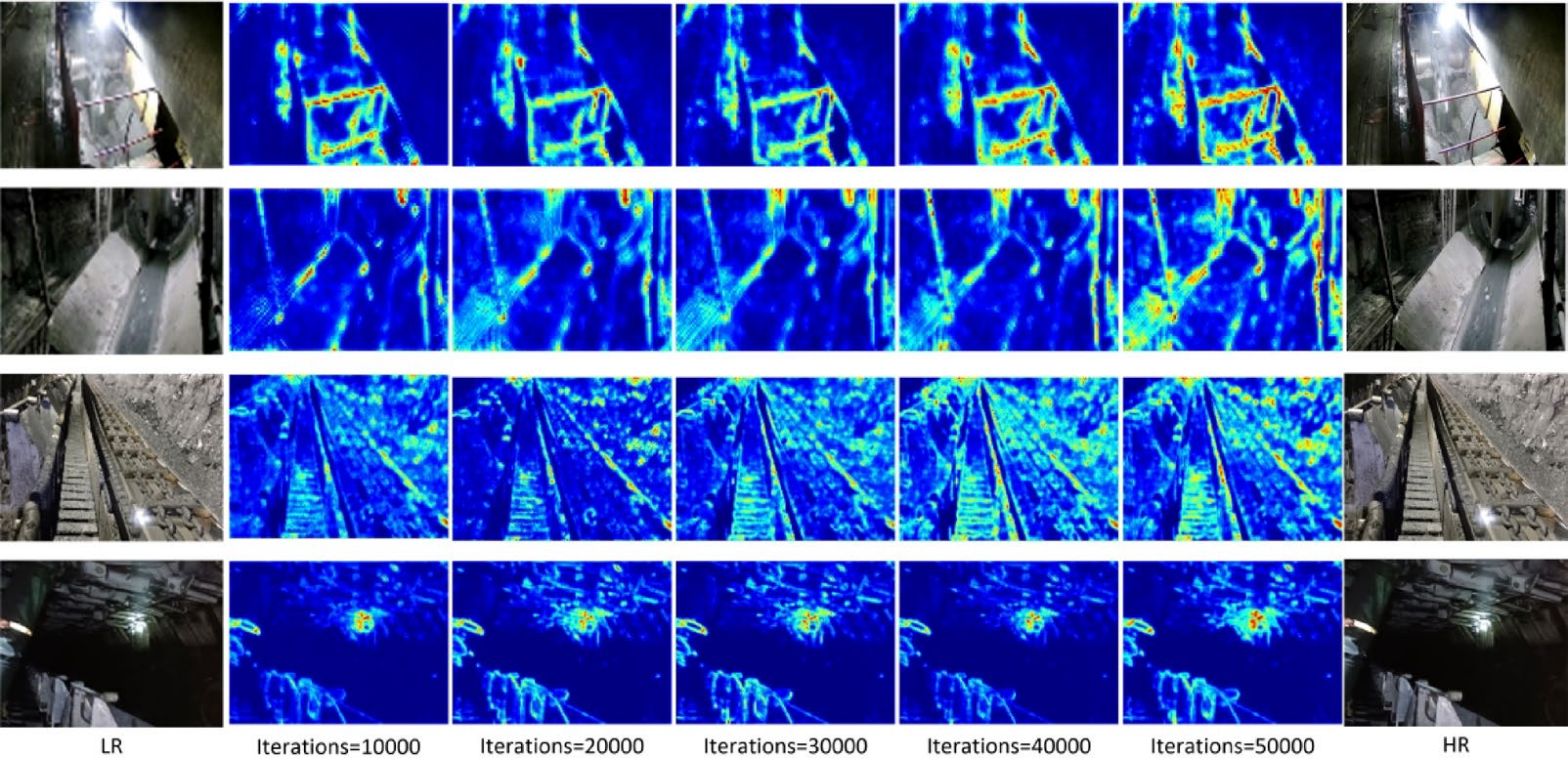
Heatmaps of the proposed method at different iteration steps.

These results suggest that LCB effectively captures local features, BAIM strengthens attention distribution, and DGFN enhances overall structural consistency. The combined effect of these modules significantly improves super-resolution reconstruction performance. Although the ablation study in Table 3 demonstrates that combining LCB, BAIM, and DGFN improves performance, it does not fully disentangle the contributions of module interaction and architectural redesign. Therefore, we conduct additional controlled experiments. First, we replace BAIM with simple addition and concatenation-based fusion to verify its effectiveness, as shown in Table 4. Second, we compare DGFN with standard FFN and DWConv-based FFN to validate the advantages of dynamic gating, as presented in Table 5. Finally, we analyze the bidirectional design by evaluating spatial-only and channel-only variants, as detailed in Table 6. The results show that each proposed component consistently outperforms its baseline counterpart, demonstrating that the performance gains stem from both effective feature interaction and improved feed-forward modelling. This validation provides a strong foundation for subsequent module integration and network architecture design. Figure 9 presents Grad-CAM feature heatmaps of the proposed method at iterations 10,000, 20,000, 30,000, 40,000, and 50,000, intuitively illustrating the differences in detail preservation and structural restoration across iterations.

To comprehensively evaluate the performance of the proposed super-resolution reconstruction method for mine images, four test datasets were selected: Set5, Set14, Urban100, and a coal mine test dataset. Experiments were conducted under 2× and 4× scaling. The experimental results are presented in Table 7.

**Table 7:** Performance comparison of different methods on public and mine datasets.

Scale	Method	Set5	Set14	Urban100	Coal mine
		PSNR/SSIM	PSNR/SSIM	PSNR/SSIM	PSNR/SSIM
	Bicubic	33.66/0.9299	30.24/0.8688	26.88/0.8403	28.12/0.9215
×2	SRCNN	36.66/0.9542	32.45/0.9067	29.50/0.8946	30.25/0.9513
	VDSR	37.53/0.9587	33.03/0.9124	30.76/0.9140	30.43/0.9586
	EDSR	38.11/0.9602	33.92/0.9195	32.93/0.9351	30.64/0.9612
	SwinIR	38.35/0.9620	34.14/0.9227	33.40/0.9393	31.05/0.9639
	DAT	38.34/0.9619	34.43/0.9247	33.54/0.9402	31.48/0.9652
	Ours	38.60/0.9630	34.30/0.9235	33.69/0.9440	32.07/0.9688
	Bicubic	28.42/0.8104	26.00/0.7027	25.96/0.6675	22.72/0.8018
×4	SRCNN	30.49/0.8628	27.50/0.7315	24.47/0.7229	24.94/0.8221
	VDSR	31.35/0.8838	28.01/0.7674	25.18/0.7524	25.36/0.8435
	EDSR	32.46/0.8968	28.80/0.7876	26.64/0.8033	26.12/0.8632
	SwinIR	32.72/0.9021	28.94/0.7904	27.07/0.8164	26.92/0.8719
	DAT	32.74/0.9013	29.02/0.7914	27.14/0.8149	27.86/0.8823
	Ours	32.95/0.9035	28.97/0.7909	27.40/0.8180	28.10/0.8836

**Fig. 10:**
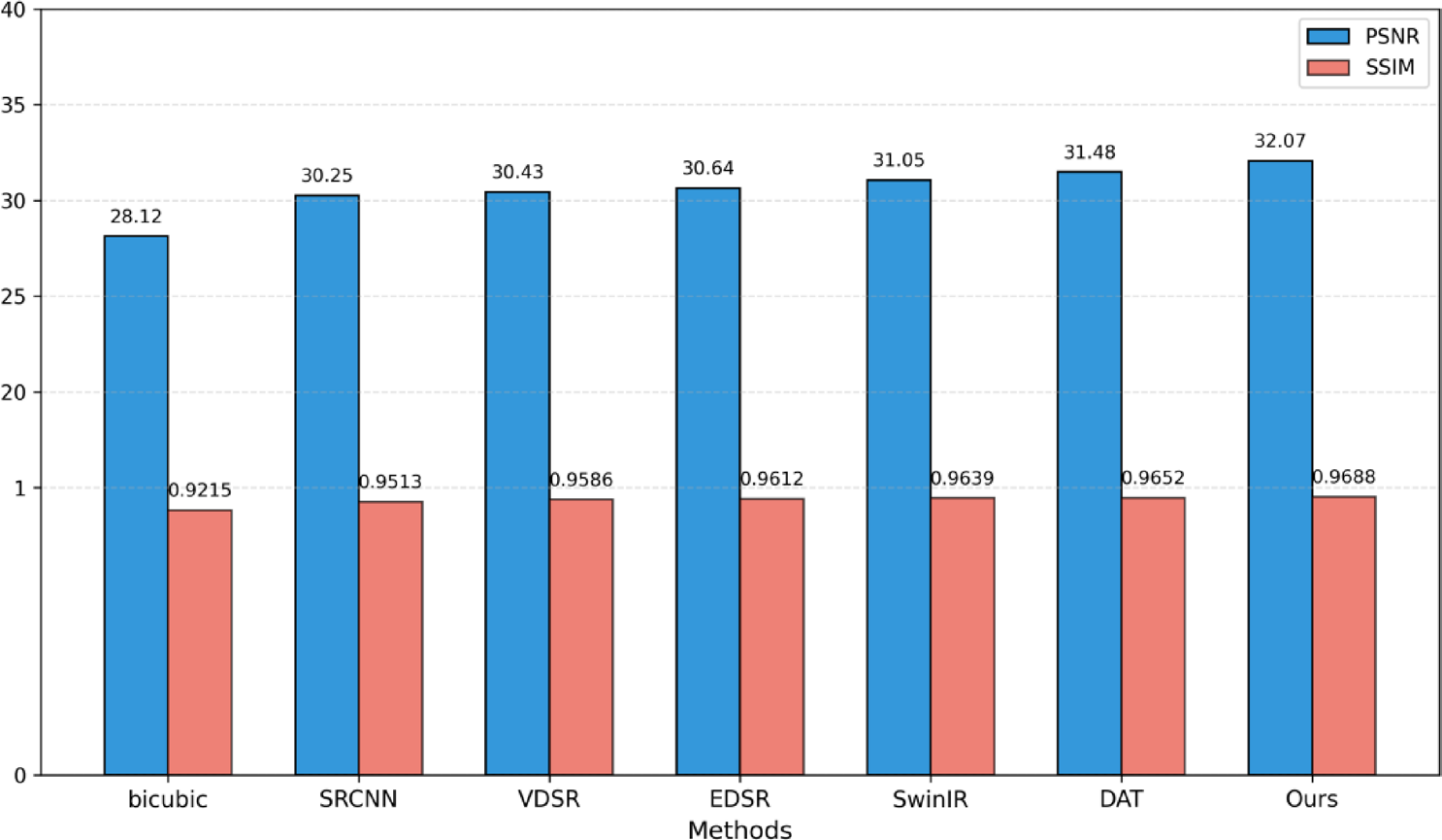
Comparison of experimental results for PSNR and SSIM with scale of 2 on the coal mine dataset.

**Fig. 11:**
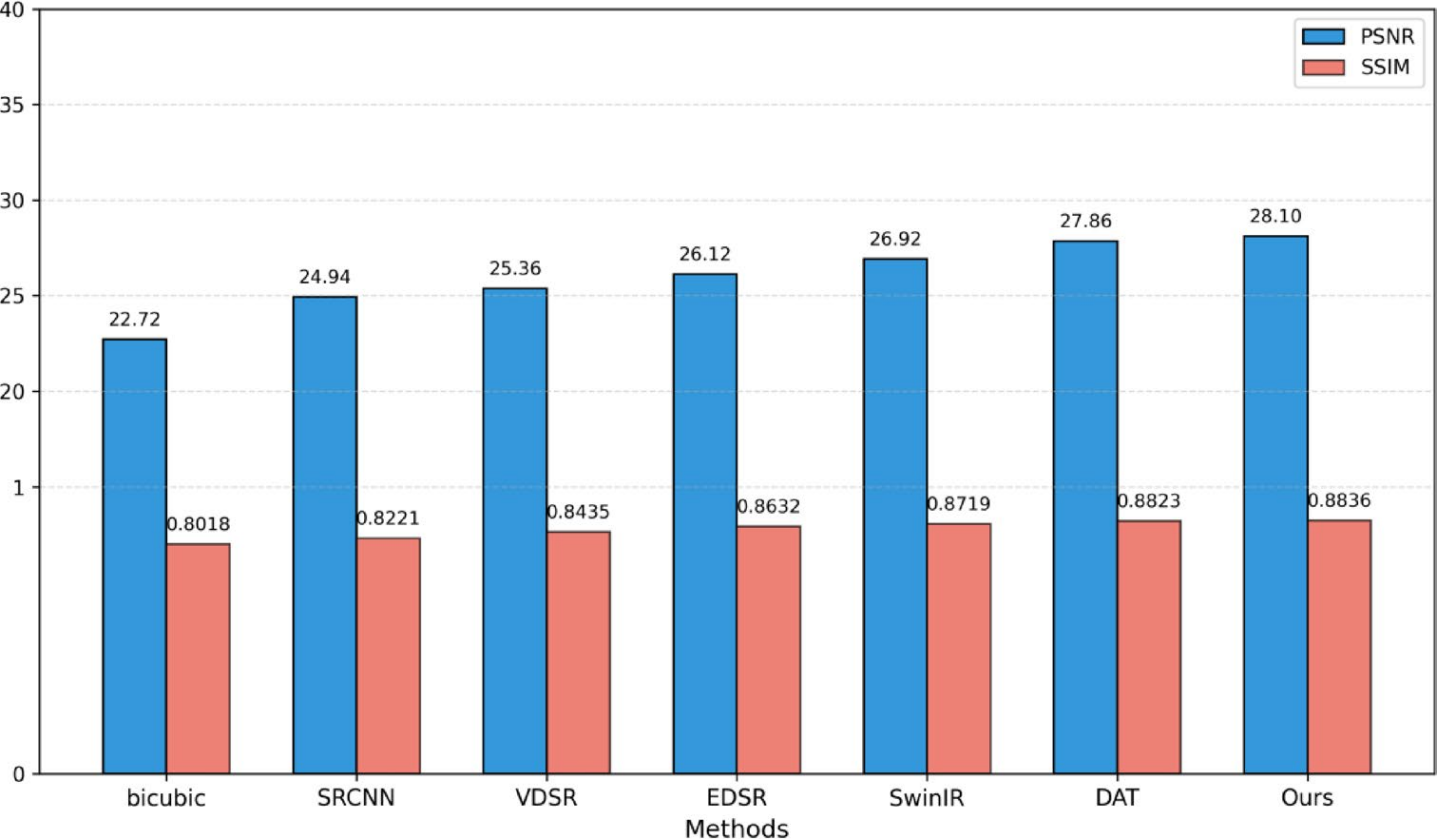
Comparison of experimental results for PSNR and SSIM with scale of 4 on the coal mine dataset.

Table 7 presents a quantitative comparison of various super-resolution reconstruction methods on the Set5, Set14, Urban100, and coal mine image datasets, covering both ×2 and ×4 scaling factors, with PSNR and SSIM as evaluation metrics. Overall, image reconstruction performance improves significantly with increased model complexity and architectural advancements. Notably, deep learning-based methods demonstrate a clear advantage over traditional interpolation techniques, especially on the coal mine dataset representing real industrial scenarios. Bar charts in Figs. 10 and 11 respectively present the PSNR and SSIM performance comparisons of mainstream super-resolution methods on the coal mine dataset at different scaling factors.

In the ×2 scale task, the traditional Bicubic method only achieves basic image reconstruction quality, while early deep learning models such as SRCNN and VDSR demonstrate significant performance improvements across all datasets. The introduction of residual learning and large-capacity network architectures in EDSR further improves PSNR and SSIM metrics compared to earlier models. Models incorporating Transformer or dynamic attention mechanisms, such as SwinIR and DAT, enhance detail restoration capabilities even further, particularly excelling on high-frequency texture-rich datasets like Urban100 and the coal mine dataset. Notably, the proposed method in this work achieves the highest or near-highest performance metrics across all four datasets, with a PSNR of 32.07 dB and an SSIM of 0.9688 on the coal mine images—representing improvements of 1.02 dB and 0.59 dB in PSNR over SwinIR and DAT, respectively. In the ×4 scale task, overall performance declines for all models, reflecting the increased difficulty of high-magnification reconstruction. However, the performance ranking remains largely consistent with the ×2 scale results. The proposed method maintains stable performance across datasets and continues to lead on the coal mine data, achieving PSNR gains of 1.18 dB and 0.24 dB over SwinIR and DAT, respectively, demonstrating strong generalization ability and robustness.

**Table 8:** Quantitative comparison of model complexity.

Method	Params (M)	FLOPs (G)	Inference Time (ms)
Bicubic	-	-	6
SRCNN	0.067	5.6	10
VDSR	0.67	40.5	31
EDSR	43.09	823.34	78
SwinIR	11.90	215.32	45
DAT	11.21	203.34	42
Ours	11.08	198.56	39

To evaluate computational efficiency, we analyzed the number of Params, FLOPs, and inference time of several representative super-resolution methods, as presented in Table 8. Although EDSR achieves high reconstruction performance, it incurs a substantially greater computational cost, with 43.09 M, 823.34 G, and an inference time of 78 milliseconds per image. Transformer-based models such as SwinIR and DAT provide a better balance between performance and efficiency, yet they still demand more computational resources than our network. The proposed method delivers comparable or superior reconstruction performance while maintaining the lowest model complexity among Transformer-based approaches, with 11.08 M, 198.56 G, and an inference time of 39 ms. These findings demonstrate that the BAIM and DGFN modules not only improve reconstruction quality but also enable efficient computation.

The proposed model demonstrates leading super-resolution reconstruction capabilities on both standard image super-resolution datasets and typical industrial scenarios. Notably, it achieves superior image quality and detail restoration in coal mine image enhancement tasks, validating its potential and effectiveness for practical applications. This success can be attributed to the model’s adaptive ability to capture the characteristic feature distribution of coal mine images, enabling more effective reconstruction of textures under complex lighting conditions and low-contrast scenes.

## Discussion

This paper addresses the problems of blur and low resolution in underground coal mine images by proposing a multi-module integrated image SR reconstruction network, and conducts systematic comparative experiments on several public datasets as well as a self-constructed coal mine dataset. Experimental results show that the proposed method outperforms classical approaches such as SRCNN, SwinIR, and DAT across multiple evaluation metrics, with particularly stable performance in PSNR and SSIM. These results demonstrate that the proposed model offers significant advantages in image detail restoration and structural preservation. Furthermore, from a subjective visual perspective, the proposed method shows superior reconstruction capability for critical structural regions such as equipment edges and texture details in coal mine images. This effectively enhances image clarity and recognizability, providing a more reliable input foundation for subsequent machine vision tasks such as object detection and recognition^46^. Ablation studies further analyzed the contributions of the LCB, BAIM, and DGFN. Results indicate that each module positively contributes to performance improvement, and their collaborative integration yields better results than individual modules alone, thereby validating the rationality and effectiveness of the overall network design.

To provide a deeper understanding of the model’s internal mechanisms and the design rationale, we conducted additional ablation experiments summarized in Tables 4, 5 and 6. Table 4 compares different feature fusion strategies, showing that BAIM significantly outperforms simple additive or concatenation-based fusion, indicating that bidirectional spatial-channel interactions effectively integrate local convolutional features with global transformer representations. Table 5 compares standard FFN, DWConv-FFN, and the proposed DGFN, demonstrating that DGFN’s dual-gated design with cross-group interactions preserves high-frequency information while balancing spatial and channel feature processing. Table 6 evaluates the contributions of the spatial and channel branches in BAIM, confirming that the full bidirectional configuration achieves the best performance. Collectively, these quantitative results provide strong evidence that the superior performance of our network arises from the thoughtful architectural design of BAIM and DGFN rather than simply increasing network depth or parameters. This analysis supports the effectiveness and rationality of each module and explains why the integrated network consistently achieves high-quality reconstruction on complex coal mine images.

Despite these achievements, several limitations remain. First, the current model architecture is relatively complex, resulting in slower inference speeds that hinder direct deployment on resource-constrained edge devices in underground environments. Second, model training mainly relies on supervised learning, requiring large amounts of high-quality paired image data, which poses challenges in acquiring high-resolution samples in real coal mine scenarios. Third, the robustness of the model under complex conditions such as image noise, occlusion, and low illumination has not yet been systematically evaluated. Future work will focus on model lightweighting, unsupervised or self-supervised training mechanisms, and enhancing model adaptability to extreme environments, aiming to further promote the practical application of coal mine image SR technology in production settings.

## Conclusion

This paper proposes a multi-module fusion network model tailored for SR reconstruction of coal mine images. By incorporating structures such as local context enhancement, edge attention mechanisms, and deep feature fusion, the model effectively improves the reconstruction quality of low-resolution underground images. Experimental results on public datasets as well as on real coal mine image datasets demonstrate that the proposed method outperforms existing mainstream algorithms in terms of PSNR and SSIM, achieving clearer restoration of image edges and texture details. This approach not only enhances the visual quality of coal mine images but also provides a solid image foundation for subsequent intelligent detection tasks.

## Data Availability

The datasets used and/or analysed during the current study are available from the corresponding author on reasonable request.
